# TSG-6 secreted by human umbilical cord-MSCs attenuates severe burn-induced excessive inflammation via inhibiting activations of P38 and JNK signaling

**DOI:** 10.1038/srep30121

**Published:** 2016-07-22

**Authors:** Lingying Liu, Huifeng Song, Hongjie Duan, Jiake Chai, Jing Yang, Xiao Li, Yonghui Yu, Xulong Zhang, Xiaohong Hu, Mengjing Xiao, Rui Feng, Huinan Yin, Quan Hu, Longlong Yang, Jundong Du, Tianran Li

**Affiliations:** 1 Department of Burn & Plastic Surgery, the First Affiliated Hospital to PLA General Hospital.

## Abstract

The hMSCs have become a promising approach for inflammation treatment in acute phase. Our previous study has demonstrated that human umbilical cord-MSCs could alleviate the inflammatory reaction of severely burned wound. In this study, we further investigated the potential role and mechanism of the MSCs on severe burn-induced excessive inflammation. Wistar rats were randomly divided into following groups: Sham, Burn, Burn+MSCs, Burn+MAPKs inhibitors, and Burn, Burn+MSCs, Burn+Vehicle, Burn+siTSG-6, Burn+rhTSG-6 in the both experiments. It was found that MSCs could only down-regulate P38 and JNK signaling, but had no effect on ERK in peritoneal macrophages of severe burn rats. Furthermore, suppression of P38 and JNK activations significantly reduced the excessive inflammation induced by severe burn. TSG-6 was secreted by MSCs using different inflammatory mediators. TSG-6 from MSCs and recombinant human (rh)TSG-6 all significantly reduced activations of P38 and JNK signaling induced by severe burn and then attenuated excessive inflammations. On the contrary, knockdown TSG-6 in the cells significantly increased phosphorylation of P38 and JNK signaling and reduced therapeutic effect of the MSCs on excessive inflammation. Taken together, this study suggested TSG-6 from MSCs attenuated severe burn-induced excessive inflammation via inhibiting activation of P38 and JNK signaling.

Severe burn is a devastating trauma with systemic consequences^1^. Severe burn triggers an excessive inflammations and serious metabolic disturbances^2^. Meanwhile, excessive inflammation is possibly an important contributor to other systemic disorders such as cardiac dysfunction, acute respiratory distress syndrome, acute renal failure, intestinal bacterial translocation, hypermetabolism, and sepsis^3^. These intense disruptions in body’s homeostatic balance will result in multiple organ failure and even death^4^. Therefore, research for seeking new treatments to attenuate excessive inflammation after severely burned injury is very necessary, especially in the acute phase.

Numerous reports have described that intravenously infused human mesenchymal stem cells (hMSCs) modulated excessive inflammation and thereby improved myocardial infarction, corneal injury, peritonitis, systemic lupus erythematosus, ulcerative colitis, or hepatic fibrosis in animal models^5^,^6^,^7^,^8^,^9^,^10^. Our previous study has shown that human umbilical cord-MSCs transplantation can accelerate wound healing of severe burn rats through alleviating the inflammatory microenvironment of locally burned wound^11^. And the beneficial effects of hMSCs were explained in part by activation of the cells to secrete a variety of bioactive factors that modulated inflammatory and immune responses, limited progression of inflammation, and enhanced tissue repair^12^,^13^. Among all the bioactive factors secreted by hMSCs, tumor necrosis factor (TNF)-a–stimulated gene/protein 6 (TSG-6) as a multipotent anti-inflammatory protein was a key natural modulator of inflammation^14^,^15^. TSG-6 was shown to modulate pro-inflammatory cytokine cascades and enhance injured tissue repair in several animal models^16^,^17^. For example, TSG-6 can suppress inflammatory reactions triggered by ischemia in the heart and thereby limit the destruction of cardiomyocytes by inhibiting neutrophils and monocytes/macrophages invading^18^. In transgenic mice, inactivation of the TSG-6 gene increased inflammatory responses and over-expression of the TSG-6 gene decreased inflammatory responses^19^,^20^. In addition, administration of the recombinant protein (rhTSG-6) largely improved arthritis and decreased inflammation in several murine models^21^,^22^.

MAPKs signaling play critical roles in regulation of inflammatory responses. The MAPKs including P38, c-Jun N-terminus kinase (JNK), and extracellular signal regulated kinase (ERK), were activated by cellular stress and proinflammatory cytokines^23^. Burned injury results in a cellular stress in the skin^24^. The P38 is activated in the skin wound after burned injury, whereas administration of P38 inhibitor in topical wound may reduce the adversely inflammatory responses^25^. Liu *et al.* showed TSG-6 secreted by mesenchymal stem cells inhibited lipopolysaccharide-induced inflammatory responses of BV2 microglial cells through inhibiting the activation of nuclear factor (NF)-κB and MAPK pathways^26^. Therefore, based on the basis of our previous study, our present study was designed to further investigate the potential mechanism involving effects of human umbilical cord-MSCs on severe burn injury-induced excessive inflammation.

## Materials and Methods

### Cell Preparations

Institutional Review Board approval was obtained from the Human Research Protection Program at the First Affiliated Hospital to PLA General Hospital for all aspects of this study. The methods described were carried out in accordance with the approved guidelines, and informed consent was obtained from all subjects. The human umbilical cord-MSCs were isolated from discarded umbilical cord donated with consensus from 5 full-term healthy fetuses were born via caesarean delivery (gestation age, 39–40 weeks), expanded *in vitro*, and characterized as previously described^27^. In the following passages, abbreviation of human umbilical cord-MSCs was MSCs. MSCs of passages 3–8 were used for all the following experiments.

### Cell treatments

MSCs were plated at 5000 cells/cm^2^ in 6-well plates with supplementation of 3 mL mesenchymal stem cell medium-serum free (MSCM-sf) (ScienCell Research Laboratories, San Diego, CA) and incubated for 1 day. The mediums were then replaced with 3 mL of MSCM-sf containing 10 ng/mL hTNF-α (Peprotech, USA), 100 ng/mL LPS (Sigma-Aldrich, USA), 10% sham serum and 10% burn serum at 24 hours post severe burn for continuous culturing. To confirm increased expression and synthesis of TSG-6, RNA and protein were extracted from aliquots of the cells and assayed for TSG-6 expression and synthesis by real-time PCR and Western blotting. The TSG-6 secreted into supernatant was detected by ELISA assay.

### Transfection with siRNA for TSG-6

 The lentiviral expression vector containing the TSG-6 siRNA sequence (GeneChem, Shanghai, China) was selected for specifically targeting TSG-6 knockdown, which was classified as Lenti-TSG-6-siRNA, and Lenti-GFP-siRNA as negative control vector. The TSG-6 siRNA lentivirus vectors were generated by ligating the vector pGC-LV. TSG-6 siRNA oligonucleotide sequences are: Forward, 5′-CCGGTTCTCCGAACGTGTCACGTTTCAAGAGAACGTGACACGTTCGGAGAATTTTTG-3′; Reverse, 5′-AATTCAAAAATTCTCCGAACGTGTCACGTTCT CTTGAAACGTGACACGTTCGGAGAA-3′. The sequences of control siRNA are: Forward, 5′-CCGGGCAAGCTGACCCTGAAGTTCATCTCGAGATGAACTTCAG GGTCACGTTGCTTTTTG-3′, Reverse, 5′-AATTCAAAAAGCAAGCTGACCCTG AAGTTCATCTCGAGATGAACTTCAGGGTCACGTTGC-3′. The recombinant lentivirus was produced by cotransfecting HEK293T cells with pGC-LV-GFP-siRNA or pGC-LV-TSG-6-siRNA, pHelper 1.0, and pHelper 2.0 plasmid using Lipofectamine 2000 (GeneChem, Shanghai, China). MSCs were transduced with the prepared lentivirus (Lenti-TSG-6-siRNA or Lenti-GFP-siRNA). MSCs were plated at 5000 cells/cm^2^ in 6-well plates or T-75 culture bottles with 3 mL or 10 mL MSCM-sf without antibiotics. After incubation for 1 day, cells were transfected with 1 ml mixture of Lenti-TSG-6-siRNA or Lenti-GFP-siRNA according to the protocol provided by the manufacturer. Twelve hours later, the medium was replaced with MSCM-sf without antibiotics, and these cells were incubated for 3–4 days sequentially^28^. After the efficiency of TSG-6 knockdown in MSCs of 6-well plates was evaluated using real-time quantitative RT-PCR and Western blot. Then MSCs in T-75 culture bottles were harvested with 0.25% trypsin, and resuspended at 5 × 10^6 ^cells in 1 mL sterile PBS for the subsequent injection.

### Animals and Reagents

All studies adhered to procedures consistent with the International Guiding Principles for Biomedical Research Involving Animals issued by the Council for the International Organizations of Medical Sciences (CIOMS.), and all experimental protocols were approved by the Institutional Animal Care and Use Committee at the First Affiliated Hospital to PLA General Hospital. Six-week-old male Wistar rats (180–220 g) were obtained from the local animal facility, housed at the Institute of Animal Experiments of the First Affiliated Hospital to PLA General Hospital in stables with a temperature of 22 °C, a relative humidity of 55% and a day/night cycle of 12/12 hours, with food and water ad libitum throughout the experiment.

### Experimental Groups

The study was made up of two animal experiments. In the first experiment, 144 Wistar rats were randomly divided into 6 groups: Sham, Burn, Burn+MSCs (burn transplanted MSCs), Burn+SB (burn transplanted with specific inhibitor of P38, SB203580), Burn+SP (burn transplanted with specific inhibitor of JNK, SP600125), Burn+PD (burn transplanted with selective inhibitor of ERK, PD098059). In the second experiment, 120 Wistar rats were randomly divided into 5 groups: Burn, Burn+MSCs, Burn+Vehicle (burn transplanted MSCs transduced with Lenti-GFP-siRNA), Burn+siTSG-6 (burn transplanted MSCs transduced with Lenti-TSG-6-siRNA), Burn+rhTSG-6 (burn administered rhTSG-6). Each group in two experimental parts was divided equally into four subgroups of 6 rats according to the time points of euthanasia at 6 h, 12 h, 24 h, 48 h after severe burn.

### Animal Model of Severely Burned Injury and Treatment

Rats were anesthetized by intraperitoneal injection of 300 mg/kg Avertin (20 mg/ml) (2,2,2-tribromoethanol, Sigma, USA)^29^. The dorsal and abdominal hair was removed completely, first with clippers and then through the application of Veet depilatory cream. Both whole backside and abdomen were placed in hot water (94 °C) for 12 s and 6 s, respectively. which caused 50% TBSA with a full-thickness burn^30^. Intraperitoneal injections of balanced salt solution (40 ml/kg) were immediately administered to prevent shock in all of groups in both experiments. Balanced salt solutions with 25 mg/kg SB203580, 30 mg/kg SP600125, 15 mg/kg PD98059 (Selleckchem, USA) were administered to the rats of Burn+SB, Burn+SP and Burn+PD groups in the first experiment, respectively^31^,^32^,^33^,^34^. The rats in the Burn+MSCs group immediately received a tail vein injection of 5 × 10^6^ MSCs immediately after burn in the both experiments. The rats in the Burn+Vehicle and Burn+siTSG-6 groups received a tail vein injection of 5 × 10^6^ MSCs transfected with Lenti-GFP-siRNA and Lenti-TSG-6-siRNA at the same time in the second experiment. The rats in the Burn and Burn+rhTSG-6 groups received a tail vein injection of 1 mL PBS and rhTSG-6 in 1 mL PBS (50 μg/mL) (Abnova, Taipei, Taiwan of China) at the same time, respectively. All rats in each group were clinically evaluated. The burn wound was then treated with 1% tincture of iodine and kept dry to prevent infection. The rats in the sham group were placed in water at 37 °C for 12 s, and the other processes were the same as those applied to the burned rats. Wounds were left open and animals were sacrificed at defined time points after burn.

### Specimen collection and detection

The blood samples were taken from aortaventralis at 6 h, 12 h, 24 h and 48 h after burn or sham burn. They were transferred immediately to heparin-containing tubes for blood routine examination and serums for the ELISA assay were collected by centrifugation. Meanwhile, samples of vital organs such as lung, kidney, heart, liver, and spleen were collected. These samples were carefully removed and rinsed in PBS. Subsequently, every organ specimen was divided into two pieces. One piece of the organ specimen was stored in liquid nitrogen for ELISA assay or future molecular detection, and another piece was fixed in 4% paraformaldehyde for HE staining and immunohistochemical examination.

To confirm regulation of MSCs/rhTSG-6 on MAPKs signals, rats in all groups were euthanized and resident macrophages in enterocoelia were harvested for western detection by washing the peritoneal cavity with 10 mL ice-cold D-Hanks solution.

### Temperature examination

At 6 h, 12 h, 24 h and 48 h after burn, rectal temperatures were measured using an electronic thermometer (OMRON MC-341, Japan) by a small mammal rectal thermister inserted 8 to 10 cm beyond the anal sphincter. The ambient temperature of the environmentally controlled room was kept at 22 ± 1 °C with a 40% to 50% relative humidity.

### Blood routine examination

The blood and serum samples were detected by the Central Institute of Clinical Chemistry and Laboratory Medicine of the First Affiliated Hospital to PLA General Hospital. A 4 mL aliquot of blood was taken for full blood count (FBC) analysis to determine all the whole blood parameters. FBC was analyzed using a Sysmex XE 2100 (Sysmex UK, Milton Keynes, UK) automated hematology analyzer within 2 hours of collection.

### Histological analyses

After fixation with 4% paraformaldehyde for 1 week at room temperature, the specimens were embedded in paraffin and sectioned in a plane perpendicular to the incision. Five-micrometer-thick sections were prepared, deparaffinized in dimethylbenzene, and rehydrated. Preparative sections were stained with H&E in accordance with standard procedures. Other sections were incubated with specific antibodies (monoclonal mouse antibodies against rat MPO, CD68; R&D Systems, Minneapolis, MN), followed by incubation with the corresponding secondary antibody and the PAP (peroxidase–anti-peroxidase) complex, and stained with DAB (3,3′-diaminobenzidine). The OD value of neutrophil (MPO+) and number of macrophage (CD68+) in the lung, liver, heart, renal and spleen tissues were counted in 5 randomly selected fields of each slide by an experienced and independent cell scientist in a blinded manner.

### GFP-fluorescence observation

To confirm successful transfection with Lenti-GFP-siRNA and Lenti-TSG-6-siRNA, we observed GFP fluorescence intensity and obtained images using the inverted fluorescence microscope (Leica, Germany).

### Real Time-PCR Analysis

After cells treatments and cells transfection with Lenti-GFP-siRNA and Lenti-TSG-6-siRNA as described previously, MSCs were resuspended in 1 ml Trizol for RNA isolation. A reverse-transcription reaction was performed to generate cDNA for all samples. The cDNA was amplified using the following gene-specific primers, according to the manufacturer’s protocol (forward and reverse, respectively): TSG-6 (208 bp), 5′-GATGGATGGCTAAGGGCAGAGT-3′ and 5′-TCATTTGGGAA GCCTGGAGATT-3′; ACTIN (317 bp), 5′-CACCCAGCACAATGAAGATCAAGA T-3′ and 5′-CCAGTTTTTAAATCCTGAGTCAAGC-3′. Real-time PCR reactions were incubated at 95 °C for 2 min, and then 40 cycles at 95 °C for 3 s followed by 60 °C for 30 s. Relative quantitation of gene expression in terms of fold change was performed using the 2^−△△CT^ method.

### Western Blotting

The treated MSCs and the peritoneal macrophages of severe rats were immediately extracted using RIPA buffer supplemented with Halt protease and a phosphatase inhibitor cocktail (Servicebio, Wuhan, China). Proteins were subjected to SDS-PAGE gel, and anti-TSG-6 antibody (1:1,000), anti-P38 and anti-P-P38 antibodies (1:2,000), anti-JNK and anti-P-JNK antibodies (1:2,000), anti-ERK and anti-P-ERK antibodies and anti-β-actin antibody (1:20,000) (R&D Systems, Minneapolis, MN) were used for protein expression assay.

### Enzyme-linked immunosorbent assay (ELISA)

The levels of tumor necrosis factor alpha (TNF-α), interleukin-1β (IL-1β), interleukin-6 (IL-6), interleukin-10 (IL-10) , myeloperoxtidase (MPO) and TSG-6 in serum/supernatant/organ specimen were detected by a multidetection microplate reader using a double-antibody sandwich ELISA kit (eBioscience, USA) according to the manufacturer’s protocols.

### Statistical analysis

All data were expressed as the mean ± SD (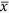 ± s) and analyzed using SPSS 18.0 (SPSS Inc., Chicago, IL, USA). Statistical differences among the groups were assessed by one-way ANOVA and after hoc multiple comparisons were performed using Student-Newman-Keuls tests. The significance level was set at p < 0.05.

## Results

### Effects of MSCs and MAPKs inhibitors on MAPKs phosphorylations

**Figure 1:**
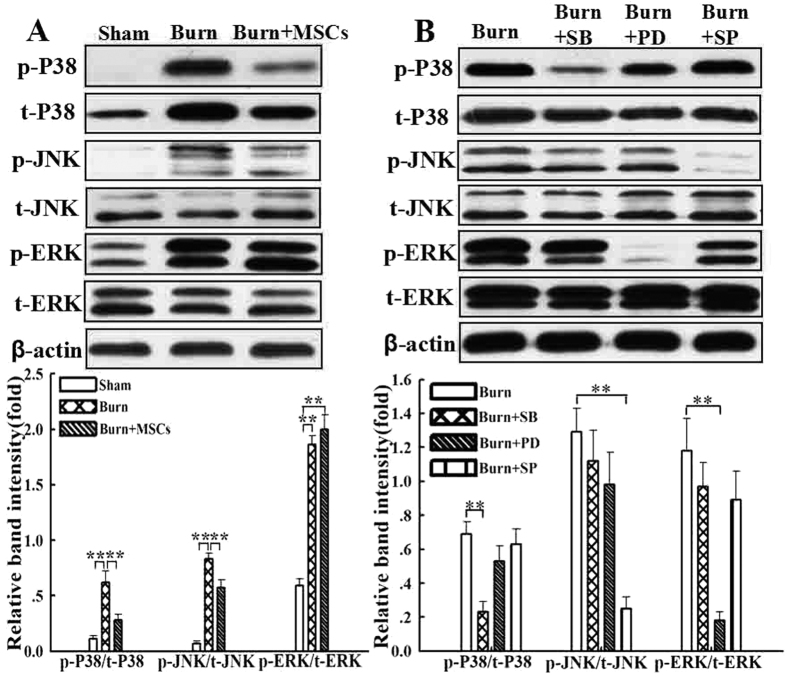
The human umbilical cord-MSCs/MAPKs inhibitors injections reduced the activations of MAPKs signaling induced by severe burn. (**A**) Intravenous (i.v.) injections of human umbilical cord-MSCs reduced levels of phosphorylated MAPKs induced by severe burn. Compared with that of sham group, the pro-inflammatory MAPKs signals including P38, JNK, and ERK of burn group were significantly activated by severe burn, and levels of phosphorylated p38 and JNK in the burn+MSCs group were down-regulated markedly, but the phosphorylation of ERK was unaffected. The quantitative analysis was reported in the histogram. (**B**) Intraperitoneal (i.p.) injection of SB203580, SP600125, and PD098059 (the specific and selective inhibitors of P38, JNK and ERK signaling) significantly decreased the corresponding phosphorylation levels of P38, JNK, and ERK after severe burn, respectively. And there was no change in total protein levels of P38, JNK, and ERK in any group. The quantitative analysis was reported in the histogram.

To investigate whether MSCs regulated the pro-inflammatory MAPKs signals (including P38, JNK, and ERK) or not, we examined the levels of phosphorylated P38, JNK, and ERK in the peritoneal macrophages at 24 h after burn by Western blotting. As shown in Fig. 1A, phosphorylated P38, JNK, and ERK in peritoneal macrophages of burn group were significantly increased compared with that of the sham group. MSCs administration markedly down-regulated the phosphorylated levels of P38 and JNK but not phosphorylated ERK (p < 0.05). In addition, as seen in Fig. 1B, the intraperitoneal injection of SB203580 prevented the burn-induced phosphorylation of P38, whereas phosphorylations of JNK and ERK were completely unaffected by SB203580. In the same way, administrations SP600125 and PD098059 also significantly decreased the corresponding levels of phosphorylated JNK and ERK, and other signals were not affected. There was no change in total protein levels of P38, JNK, and ERK in any group.

### Suppression of P38 and JNK activations reduced the systemic inflammatory responses induced by severe burn

**Figure 2:**
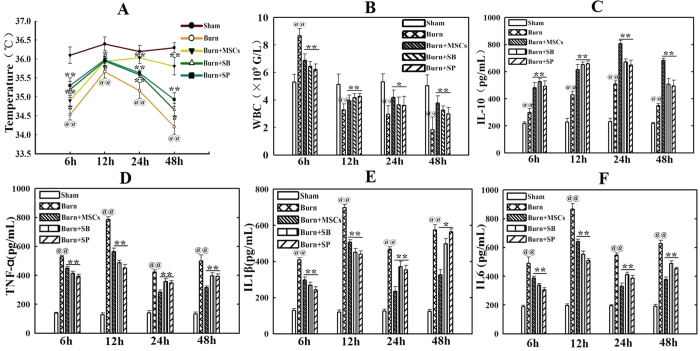
Suppression of p38 and JNK activations reduced the systemic inflammatory responses induced by severe burn. (**A**) Administrations of human umbilical cord-MSCs and P38, JNK inhibitors significantly improved hypothermia induced by severe burn at 6 h, 12 h, 24 h and 48 h post burn. (**B**) Administrations of human umbilical cord-MSCs and P38, JNK inhibitors significantly improved leukocyte counts to close to the normal level at 6 h, 12 h, 24 h and 48 h after severe burn. (**C–F**) Administrations of human umbilical cord-MSCs and P38, JNK inhibitors markedly increased level of anti-inflammatory cytokine of IL-10 and decreased levels of pro-inflammatory cytokines, including TNF-α, IL-1β, IL-6 in serum after severe burn. Values are represented as mean ± SD (n = 6). A single (^@^) and double (^@@^) stand for p < 0.05 and P < 0.01 compared with sham group, respectively. Asterisk (*) and double asterisk (**) stand for p < 0.05 and p < 0.01 compared with burn group, respectively.

To investigate influence of inhibiting the activations of P38 and JNK signalings on the systemic inflammatory responses induced by severe burn, we assayed temperature, leukocyte counts and levels of pro-inflammatory and anti-inflammatory cytokines at 6 h, 12 h, 24 h, and 48 h after severe burn. As shown in Fig. 2A, compared with sham group, severe burn-induced hypothermia and temperature of burned rats was lower than 36 °C at all time points. However, MSCs, SB203580, and SP600125 injections significantly improved hypothermia induced by severe burn. Temperature in burn+MSCs group was even close to normal value at 24 h and 48 h after injection. Likewise, MSCs, SB203580, and SP600125 injections significantly improved leukocyte counts, which were less than 4 × 10^9^/L at 12 h, 24 h and 48 h after severe burn, to close to the normal value (Fig. 2B). It was also found that these injections markedly increased levels of the anti-inflammatory cytokines IL-10 and decreased levels of the pro-inflammatory cytokines such as TNF-α, IL-1β, IL-6 in serum at 6 h, 12 h, 24 h, 48 h after severe burn (Fig. 2C–F).

### Suppression of P38 and JNK activations reduced the inflammatory cells infiltrations in vital organs after severe burn

**Figure 3:**
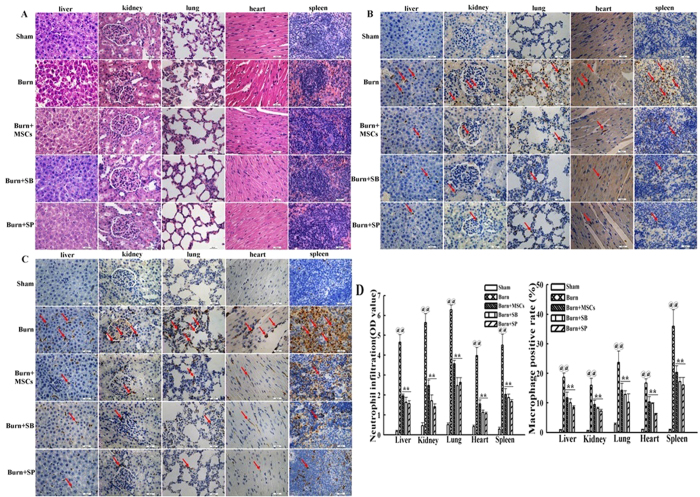
Suppression of p38 and JNK activations reduced the inflammatory cells infiltrations in vital organs at 24 h post severe burn. (**A**) Administrations of human umbilical cord-MSCs and P38, JNK inhibitors markedly reduced infiltration degrees of total inflammatory cells in liver, kidney, lung, heart, and spleen after severe burn. The infiltration degrees of total inflammatory cells were assessed by H&E staining (The light microscopy, 400×). (**B**) Administrations of human umbilical cord-MSCs and P38, JNK inhibitors significantly reduced the neutrophil infiltrations in liver, kidney, lung, heart, and spleen after severe burn. Positive staining for neutrophils (MPO) was examined by immunohistochemistry (The light microscopy, 400×). (**C**) Administrations of human umbilical cord-MSCs and of P38, JNK inhibitors markedly reduced macrophages infiltrations in liver, kidney, lung, heart, and spleen after severe burn. Positive staining for macrophage (CD68) was examined by immunohistochemistry (The light microscopy, 400×). (**D**) Quantitative analyses of positive staining for neutrophils (MPO) and macrophages (CD68) are shown in the corresponding histograms. Values are represented as mean ± SD (n = 6). A single (^@^) and double (^@@^) stand for p < 0.05 and p0.01 compared with sham group, respectively. Asterisk (*) and double asterisk (**) stand for p < 0.05 and p < 0.01 compared with burn group, respectively.

To investigate influence of inhibiting the activations of P38 and JNK signaling on the inflammatory cells infiltrations in vital organs at 24 h post severe burn, we evaluated the infiltrated degrees of the inflammatory cells in liver, kidney, lung, heart, and spleen of all groups using histochemical staining. As shown in Fig. 3A, compared with that of sham group, the infiltrated degrees of total inflammatory cells in these vital organs of burn group were remarkably increased, and they were markedly decreased by MSCs and SB203580 and SP600125 injections (p < 0.05). Further, the data showed that neutrophil (MPO+) and macrophage (CD68+) infiltrations in liver, kidney, lung, heart, and spleen were also markedly increased after severe burn, and their infiltrations were significantly decreased by MSCs and SB203580 and SP600125 injections (p < 0.05) (Fig. 3B,C). The quantitative analysis were reported in the corresponding histogram (Fig. 3D).

### Suppression of P38 and JNK activations reduced levels of the inflammatory cytokines in vital organs after severe burn

**Figure 4:**
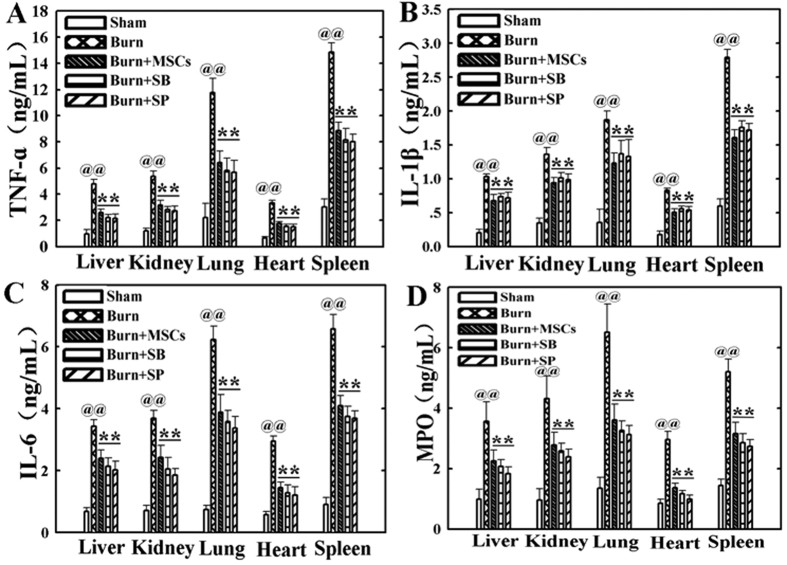
Suppression of p38 and JNK activations reduced levels of the inflammatory cytokines in vital organs at 24 h post severe burn. (**A**) Administrations of human umbilical cord-MSCs and P38, JNK inhibitors significantly reduced levels of TNF-α in liver, kidney, lung, heart, and spleen post severe burn. (**B–D**) Compared with that of sham group, the levels of IL-1β, IL-6 and MPO in these vital organs of burn group were remarkably increased, and they were significantly decreased by human umbilical cord-MSCs and P38, JNK inhibitors administrations. Values are represented as mean ± SD (n = 6). A single (^@^) and double (^@@^) stand for p < 0.05 and p < 0.01 compared with sham group, respectively. Asterisk (*) and double asterisk (**) stand for p < 0.05 and p < 0.01 compared with burn group, respectively.

To investigate direct effect of inhibiting the activations of P38 and JNK signaling on the inflammatory cytokines levels of vital organs at 24 h post severe burn, we evaluated the levels of TNF-α, IL-1β, IL-6 and MPO in liver, kidney, lung, heart, and spleen of all groups using ELISA assay. As shown in Fig. 4A, compared with that in sham group, the levels of TNF-α in these vital organs of burn group were remarkably increased, and they were significantly decreased after injection of MSCs, SB203580 and SP600125 (p < 0.05). Similarly, the levels of IL-1β, IL-6 and MPO in these vital organs of burn group were also remarkably increased compared to those in sham group, and they were obviously reduced by MSCs, SB203580 and SP600125 administrations (p < 0.05) (Fig. 4B–D).

### TSG-6 secretion and its function on regulating P38 and JNK activations

**Figure 5:**
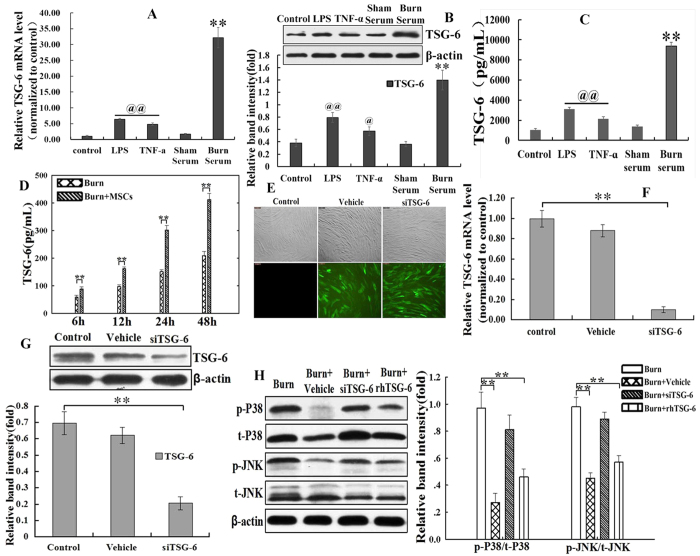
TSG-6 secretion and its function on regulating p38 and JNK activations. (**A**) The mRNA levels of TSG-6 in human umbilical cord-MSCs treated by LPS, TNF-α, sham serum and burn serum were detected by real time-PCR. (**B**) The TSG-6 protein levels in human umbilical cord-MSCs treated by LPS, TNF-α, sham serum and burn serum were detected by Western blotting. A quantitative analysis of the relative band intensity is shown in the corresponding histogram. (**C**) TSG-6 level in supernatant was evaluated by ELISA. The mRNA, protein and secretion levels of TSG-6 in the LPS and TNF-α groups were markedly increased compared with that in control group, and TSG-6 level in burn serum group were also significantly increased compared with that in sham serum. The TSG-6 level in burn serum group were the highest in all treatment groups, and its mRNA level was up to approximately 19-fold relative to that of sham serum group (**D**) The level of TSG-6 in burn serum was also evaluated using ELISA. TSG-6 level of the burn+MSCs group was significantly higher than that of burn group. (**E**) Representative photographs demonstrated successful transfection with Lenti-GFP-siRNA (vehicle) and Lenti-TSG-6-siRNA (siTSG-6) in human umbilical cord-MSCs (The inverted fluorescence microscope, 100×). (**F,G**) The levels of TSG-6 mRNA and protein expression in human umbilical cord-MSCs after transfection Lenti-GFP-siRNA and Lenti-TSG-6-siRNA were detected by real time-PCR and western blotting. (**H**) The regulating effect of TSG-6 on p38 and JNK activations. Compared with that of burn group, the levels of phosphorylated P38 and JNK signalings of burn+vehicle and burn+rhTSG-6 groups were significantly decresed, and the levels of burn+siTSG-6 group decreased slightly, but no statistically significant (P > 0.05). Values are represented as mean ± SD (n = 6). A single (^@^) and double (^@@^) stand for p < 0.05 and p < 0.01, respectively. Asterisk (*) and double asterisk (**) stand for p < 0.05 and p < 0.01, respectively.

To elucidate the potential mechanisms responsible for the effects of MSCs on anti-inflammatory properties, we next tested the levels of TSG-6 gene/protein expressions and secretion, and its function on regulating P38 and JNK activations. Data showed that the levels of mRNA, protein expressions and secretion of TSG-6 in MSCs after treatments with TNF-α, LPS and burn serum were significantly increased *in vitro* (Fig. 5A–C). It is worth noting that TSG-6 mRNA, protein and secretion levels of burn serum group were the highest in all treatment groups, and its mRNA level was up to approximately 19-fold relative to that of sham serum group (Fig. 5A–C). In animal experiment, TSG-6 in serum of the burn+MSCs group was also significantly higher than that of burn group (Fig. 5D).

As shown in Fig. 5E, representative photographs demonstrated successful transfections with Lenti-GFP-siRNA and Lenti-TSG-6-siRNA. The efficiency of TSG-6 knockdown was approximately 85% by real time-PCR (Fig. 5F). Similar results were obtained by Western blotting for TSG-6 (Fig. 5G).

As shown in Fig. 5H, compared with that of burn group, the levels of phosphorylated P38, and JNK signalings of burn+vehicle and burn+rhTSG-6 groups were significantly decreased (p < 0.05). However, the levels of phosphorylated P38, and JNK signaling in burn+siTSG-6 group had no statistically significant compared to burn group (P > 0.05).

### The effect of TSG-6 level on the systemic inflammatory responses induced by severe burn

**Figure 6:**
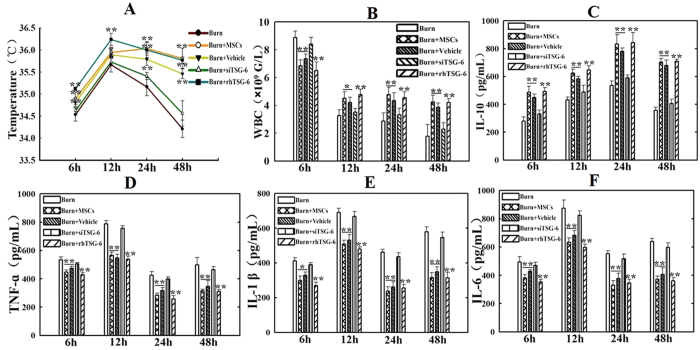
The effects of TSG-6 level on the systemic inflammatory responses induced by severe burn. (**A,B**) Administrations of human umbilical cord-MSCs with TSG-6 siRNA knockdown did not improve temperature and leukocyte counts. However, rhTSG-6 administration significantly improved temperature and leukocyte counts, which were closed to normal. (**C–F**) Administrations of human umbilical cord-MSCs with TSG-6 siRNA knockdown significantly reduced the therapeutic effect of the human umbilical cord-MSCs on anti-inflammatory cytokine of IL-10 and pro-inflammatory cytokines of TNF-α, IL-1β, IL-6 in serum after severe burn, but rhTSG-6 administration greatly improved the levels of the pro-inflammatory and anti-inflammatory cytokines. Values are represented as mean ± SD (n = 6). A sterisk (*) and double asterisk (**) stand for p < 0.05 and p < 0.01 compared with burn group, respectively.

To further evaluate the role of TSG-6 in the effect of MSCs on severe burn-induced excessive inflammation, we assayed effects of MSCs with knockdown TSG-6 and rhTSG-6 injections on temperature, leukocyte counts, and levels of pro- inflammatory and anti-inflammatory cytokines after severe burn. It was revealed that injection of MSCs with knockdown TSG-6 slightly improved temperature and leukocyte counts at 6 h, 12 h, 24 h, 48 h post severe burn, and no statistically significant (p > 0.05). But rhTSG-6 administration significantly improved temperature and leukocyte counts compared to burn group (p < 0.05) (Fig. 6A,B). Furthermore, the therapeutic effects of the MSCs on anti-inflammatory cytokines of IL-10 and pro-inflammatory cytokines, such as TNF-α, IL-1β, IL-6 in serum after severe burn were significantly reduced by MSCs with knockdown TSG-6. However, rhTSG-6 administration greatly improved levels of these inflammatory cytokines. (Fig. 6C–F).

### The effect of TSG-6 level on the inflammatory cells infiltrations induced by severe burn

**Figure 7:**
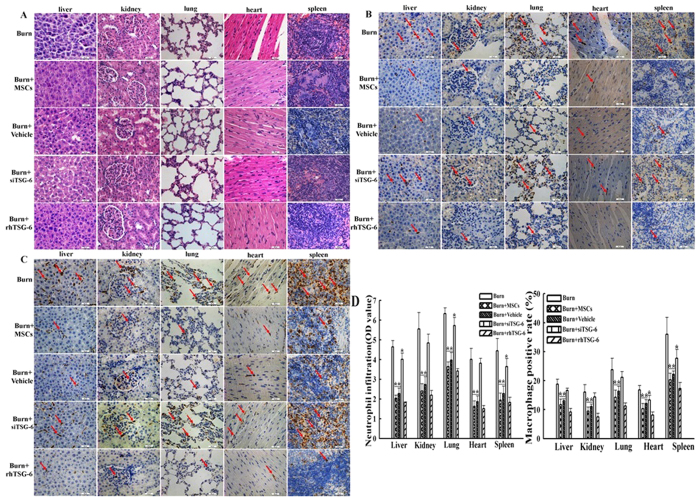
The effects of TSG-6 level on the inflammatory cells infiltrations induced by severe burn. (**A–C**) The inhibition effects of human umbilical cord-MSCs on infiltration degrees of total inflammatory cells, neutrophils, macrophages in liver, kidney, lung, heart, and spleen were significantly reduced after TSG-6 siRNA knockdown, and rhTSG-6 administration got a good anti-inflammatory effects (The light microscopy, 400×). (**D**) Quantitative analysis of positive staining for neutrophils (MPO) and macrophages (CD68) were shown in the corresponding histograms. Values are represented as mean ± SD (n = 6). A sterisk (*) and double asterisk (**) stand for p < 0.05 and p < 0.01 compared with burn group, respectively.

Next, we further assayed the influence of MSCs with knockdown TSG-6 and rhTSG-6 injections on infiltrations of total inflammatory cells, neutrophils, and macrophages in liver, kidney, lung, heart and spleen at 24 h post severe burn. Compared with that in burn group, the infiltration degrees of total inflammatory cells, neutrophils, macrophages in these vital organs in groups of the burn+MSCs, burn+Vehicle and burn+rhTSG-6 were markedly decreased (p < 0.05). Meanwhile, the infiltration degrees of these inflammatory cells in these vital organs of burn+siTSG-6 group were slightly decreased compared to burn group, but they were remarkable increased compared with that of burn+MSCs group. Furthermore, the ameliorative effect of these inflammatory cells infiltration degrees in the burn+rhTSG-6 group were approximately the effects of burn+MSCs group (Fig. 7A–C). The quantitative analysis was reported in the corresponding histogram (p < 0.05) (Fig. 7D).

### The effect of TSG-6 level on expression of the inflammatory cytokines in the vital organs induced by severe burn

**Figure 8:**
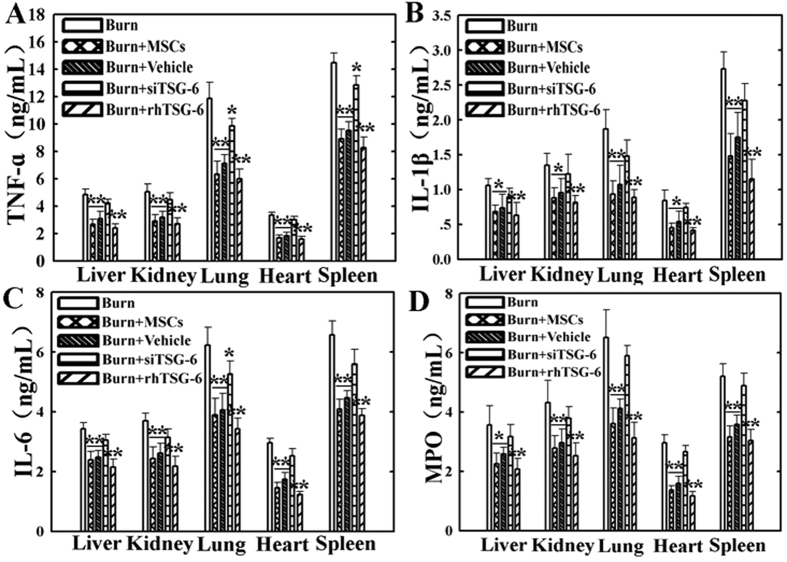
The effects of TSG-6 level on expression of the inflammatory cytokines in vital organs at 24 h post severe burn. The therapeutic effects of human umbilical cord-MSCs on levels of the inflammatory cytokines, such as TNF-α (**A**), IL-1β(**B**), IL-6 (**C**) and MPO (**D**) in liver, kidney, lung, heart, and spleen were significantly decreased after TSG-6 siRNA knockdown, and rhTSG-6 administration got a good anti-inflammatory effects for these inflammatory cytokines in vital organs. Values are represented as mean ± SD (n = 6). A sterisk (*) and double asterisk (**) stand for p < 0.05 and p < 0.01 compared with burn group, respectively.

Finally, we further analyzed the influence of MSCs with knockdown TSG-6 or rhTSG-6 injections on levels of the inflammatory cytokines, such as TNF-α, IL-1β, IL-6 and MPO in liver, kidney, lung, heart, and spleen of all groups at 24 h post severe burn. Compared with that of burn group, the levels of TNF-α, IL-1β, IL-6 and MPO in these vital organs in groups of the burn+MSCs, burn+Vehicle and burn+rhTSG-6 were also significantly decreased (p < 0.05), and they were slightly decreased in burn+siTSG-6 group (p > 0.05). In addition, we found that the therapeutic effect of MSCs on inflammatory cytokines levels in vital organs were heavily reduced after TSG-6 knockdown, but rhTSG-6 administration got a good anti-inflammatory effects, even approximately the therapeutic effects of MSCs (Fig. 8A–D).

In short, these data showed that anti-inflammatory protein TSG-6 from MSCs in the effect of the cells on severe burn-induced excessive inflammation played a key role.

## Discussion

In recent years, human umbilical cord-MSCs can be considered the perfect candidates for cell-based therapies and regenerative medicine. Human umbilical cord-MSCs can repair the damaged tissues and modulate immune systems^35^. It has been demonstrated that hMSCs are able to attenuate the inflammatory reaction of systemic lupus erythematosus, ulcerative colitis, hepatic fibrosis, and other diseases^6^,^10^,^36^,^37^. In this study, we also found that human umbilical cord-MSCs administration could significantly reduce severely burned injury-induced the excessive inflammation, including hypothermia, low numbers of leukocyte counts, undesirably high levels of pro-inflammatory cytokines and inflammatory cells infiltrations in vital organs, which were necessary diagnostic criteria of excessive inflammation^30^. On this basis, we further investigated the potential anti-inflammatory mechanisms of human umbilical cord-MSCs.

After severe burn, immune defense system from organism was initiated, subsequently a large number of neutrophils and macrophages were recruited and activated. Pro-inflammatory cytokines such as TNF-α, IL-1, and IL-6 from the hyperactive macrophages through activating internal pro-inflammatory signaling pathways, which in turn activated macrophages, resulting in a pro-inflammatory cascade reactions. Activated macrophages are a major source of inflammation related molecules^38^,^39^. Therefore, peritoneal macrophage from severely burned rats was chosen as study object to elucidate direct pro-inflammatory and anti-inflammatory mechanisms.

The MAPKs pathways, activated by cellular stress and proinflammatory cytokines, play critical roles in regulation of inflammatory responses^40^. The P38 signaling was activated in the skin wound after burned injury, and inhibiting P38 in topical wound could reduce the adverse inflammatory responses^25^. Other studies reported that inhibiting the activation of MAPKs signaling can significantly reduce inflammation and improve histological and functional outcomes of severe trauma^40^,^41^,^42^. For example, MAPK/NF-κB signaling pathways were activated in the brain after traumatic brain injury (TBI) contributing to neuronal death, and inhibition of MAPKs activation may have decreased adverse inflammatory response events and reduced the loss of neuronal cells after TBI^43^,^44^. Our study also showed that MAPKs signaling were activated immediately after severe burn, but human umbilical cord-MSCs only down-regulated levels of phosphorylated P38, and JNK after severe burn, and the level of phosphorylated ERK was unaffected. In addition, suppression of P38 and JNK activations significantly reduced excessive inflammation, including the systemic inflammatory responses, inflammatory cells infiltrations and the inflammatory cytokines levels, which were induced by severe burn in vital organs. The above results suggested that human umbilical cord-MSCs attenuated severe burn-induced excessive inflammation via inhibiting pro-inflammatory P38 and JNK signaling but not ERK.

Recent investigations have shown that MSC paracrine signaling is the primary mechanism accounting for the beneficial effects of MSC in response to injury^45^,^46^. The bioactive factors of nitric oxide synthase, indoleamine 2,3-dioxygenase, prostaglandin E2, and TSG-6 secreted by MSCs in response to tissue injury got good anti-inflammatory effects^13^,^47^,^48^. The synthesis and secretion of TSG-6 in MSCs were induced by the pro-inflammatory cytokines^18^,^35^. Transplanted MSCs played a crucial role in the suppression of inflammation in models of myocardial infarction and corneal injury, and these anti-inflammatory effects may be attributable to the secretion of TSG-6 by MSCs^5^. In this study, human umbilical cord-MSCs were activated by burn serum and soluble inflammatory mediators such as LPS and TNF-α to express and secrete TSG-6 *in vitro* and *in vivo*. Furthermore, TSG-6 levels in burn serum group were the highest in all of the treatment groups *in vitro*. That maybe because burn serum contained multiple stimulating factors, including inflammatory cytokines, burned factors, enzymes, and other factors, which induced TSG-6 expression and secretion. Most importantly, TSG-6 level in serum of the burn+MSCs group was significantly higher than that of burn group *in vivo*. The above data immediately indicated that TSG-6 from human umbilical cord-MSCs might play a very key role in the effect of the cells on severe burn-induced excessive inflammation.

We further evaluated TSG-6 in the effect of human umbilical cord-MSCs on severe burn-induced excessive inflammation, and observing influence of MSCs with knockdown TSG-6 and rhTSG-6 injections on the pro-inflammatory signals, systemic inflammatory responses, inflammatory cells infiltrations and inflammatory cytokines levels in vital organs. Data revealed that human umbilical cord-MSCs and rhTSG-6 administrations significantly suppressed severe burn-induced activations of P38, and JNK signaling, which subsequently reduced excessive inflammations. On the contrary, MSCs with TSG-6 knockdown significantly increased levels of phosphorylated P38, and JNK signaling and also reduced the therapeutic effect of the human umbilical cord-MSCs on excessive inflammation. These comprehensive data from the pro and con two aspects showed that TSG-6 from human umbilical cord-MSCs attenuates severe burn-induced excessive inflammation via inhibiting the activations of P38 and JNK signaling pathways. In addition, some studies also showed that the anti-inflammatory proteins from hMSCs could inhibit inflammations induced by some diseases or trauma through inhibiting activations of pro-inflammatory signaling^6^,^23^,^49^. A study indicated that rhTSG-6 administration also could attenuate acute lung injury-induced inflammatory response and down-regulate the pro-inflammatory signaling activations^50^.

## Conclusions

In summary, these data suggest that anti-inflammatory protein TSG-6 secreted by human umbilical cord-MSCs attenuates excessive inflammation in rats after severe burn injury via inhibiting the activations of P38 and JNK signaling pathways. Moreover, these data might provide the theoretical foundation for further clinical applications of human umbilical cord-MSC in burn areas.

## Additional Information

**How to cite this article**: Liu, L. *et al.* TSG-6 secreted by human umbilical cord-MSCs attenuates severe burn-induced excessive inflammation via inhibiting activations of P38 and JNK signaling. *Sci. Rep.* **6**, 30121; doi: 10.1038/srep30121 (2016).
